# Injury amongst Medicolegal Cases in Department of Forensic Medicine of a Tertiary Care Centre: A Descriptive Cross-sectional Study

**DOI:** 10.31729/jnma.7063

**Published:** 2021-12-31

**Authors:** Arbin Shakya, Jenash Acharya, Sunil Kumar Joshi

**Affiliations:** 1 Department of Forensic Medicine, Karnali Academy of Health Sciences, Jumla, Karnali, Nepal; 2 Department of Forensic Medicine, Kathmandu Medical College and Teaching Hospital, Sinamangal, Kathmandu, Nepal; 3 Department of Community Medicine, Kathmandu Medical College and Teaching Hospital, Kathmandu, Nepal

**Keywords:** *documentation*, *forensic medicine*, *injuries*, *Nepal*, *violence*

## Abstract

**Introduction:**

Victim of injuries presenting to a hospital is a medico-legal issue. So, with medical management, proper documentation of injuries should be done as a legal duty by all physicians attending such cases. The study aims to find the prevalence of injury amongst medicolegal cases in the Department of Forensic Medicine in a tertiary care centre.

**Methods:**

A descriptive cross-sectional study was done amongst 328 medicolegal cases presenting at a tertiary center, from January 2019 to February 2021. Ethical approval was obtained from the Institutional Review Committee (Reference number: 2603202101). Convenience sampling was used to select study samples. After detailed history regarding the incidence, injuries were examined and documented in a performa. The data were entered in Statistical Package for Social Sciences version 18. Point estimate at 95% Confidence Interval was calculated along with frequency and percentage for binary data.

**Results:**

Among 328 cases presenting to hospital for medicolegal issues, 237 (72.25%) (67.40-77.09 at 95% Confidence Interval) had injuries, out of which 170 (71.73%) cases were due to physical assault and 64 (27%) cases due to accident; the rest two (1.26%) were undetermined. Majority of victims of injury were adult males, with a mean age of 32.41±13.96 years. In most accidental injuries internal organs were also injuries and were life-threatening.

**Conclusions:**

The prevalence of injuries amongst medicolegal cases was found to be higher in our study in comparison to other studies done in similar settings. Most of the injuries were due to physical assault; however, the majority of road traffic injuries were life-threatening. These road traffic injuries could have been prevented by following a safe system approach to road safety.

## INTRODUCTION

Medico-legal examination is understood as a clinical examination of survivors of physical, sexual or mental trauma to analyze severity, mechanism, and circumstances, to document and produce reports for perusal in the administration of justice. Criminal Code of Nepal 2074 has explained injury as any harm caused due to act of commission or omission resulting in damage to an individual's body, mind, dignity, right, or a breach in contract.^1^

The fact that trauma is one of the leading causes of death and disability around the globe, and besides the World Health Organization's remarks that injury and violence have been neglected from the global health agenda for many years, despite being predictable and largely preventable; very few studies have been carried out in Nepal on trauma cases that visit hospitals for treatment.^2,3^

The study aims to find the prevalence of injury amongst medicolegal cases in Department of Forensic Medicine in a tertiary care centre.

## METHODS

A descriptive cross-sectional study was conducted among 237 victims of trauma presenting to the Department of Forensic Medicine of a Medical College and Teaching Hospital based in Kathmandu for documentation of injury for the medico-legal purpose from January 2019 to February 2021. Ethical approval was obtained from the Institutional Review Committee of the same institution (Ref no: 2603202101). Informed consent was taken from the participants, and in the case of minors, consent was taken from their guardians.

All the victims with a history of any form of trauma who were referred to the Department of Forensic Medicine by the investigating police officer for the medicolegal injury report were included in the study. The convenience sampling method was used for selecting the study participants who presented during the study period and consenting to participate in the study. The sample size was calculated using the given formula:

n = Z^2^ × p × q / e^2^

  = (1.96)^2^ × 0.50 × (1-0.50) / (0.06)^2^

  = 267

Where,

n= minimum required sample size
Z= 1.96 at 95% Confidence Interval (CI)
p= prevalence for maximum sample size calculation, 50%
q= 1-p
e= margin of error, 6%

However, we included 328 cases presented to the department for documentation of for a medico-legal purpose.

A detailed history was taken from patients or their relatives in Compos Mentis. Physical examination was performed and details of injuries present on the body were documented. Operation theatre notes, X-ray, and CT findings were included in cases where internal injuries were present. Study variables like age, sex, geographical distribution, type of injury, severity of the injury, causative weapon or force used, site of injury, circumstances or manner of causation, the relation between victim and assailant, use of any drugs/alcohol during the incident, and the cause behind the violence were recorded in Performa during data collection, the information gathered were also used as standard procedure for preparation of medico-legal injury reports.

Data were entered and analyzed using Statistical Package for Social Sciences version 18. Point estimate at 95% Confidence Interval was calculated along with frequency and percentage for binary data.

## RESULTS

Among 328 cases presenting to hospital for medicolegal issues, 237 (72.25%) (67.40-77.09 at 95 % Confidence Interval) had injury. Out of 237 injury cases, 187 (78.9%) were male and 50 (21.1%) were female, with a male to female ratio of 3.7:1. The mean age of the victims that visited for medico-legal injury examination was 32.41±13.96 years, with the youngest individual being 2years and the oldest being 91 years old. The majority of the sample population were a permanent denizen of Kathmandu district 135 (59.7%), followed by surrounding Bhaktapur (5.8%), Kavrepalanchowk (5.3%), and Ramechhap (4.6%).

Out of 237 cases of injuries, 151 (63.7%) did not require hospital admission and were discharged after initial management in the emergency department and later followed up in the outpatient department, 42 (17.7%) were admitted in general wards, 38 (16.5%) were managed in ICU and 4 (1.7%) in High dependency Unit depending upon the severity of their injury.

The majority of individuals sustained a combination of two or more injuries,108 (45.6%) compared to isolated injuries like superficial abrasions (15%), lacerations (9.7%), and sharp force injuries (2% incised, stab, and chop wound. The combination of abrasion and contusion was common findings seen as a combination.

Out of 237 cases examined, 170 cases came with a history of physical assault and 64 cases were alleged as a result of an accident (Table 1). Self-inflicted, spontaneous, and suspicious cases were nominal due to underreporting in a police station for procurement of injury reports.

**Table 1:** Circumstances of Injury (n= 237).

Circumstance	n (%)
Assault	170 (71.7)
Accident	64 (27)
Suspicious	1 (0.4)
Unknown	2 (0.8)

Injuries associated with single or multiple fractures of underlying bones were seen in 76 (32.07%) individuals. The most common fracture was bones of lower limb amounting to 23 (9.70%), followed by upper limbs, facial and cranial bones. These fractures occurred more commonly in accidental injuries (60.93%). Isolated fracture of lower limbs was common in accidental injuries whereas isolated fractures of facial bones were more common in the physical assault; as evident descriptively in cross-tabulation (Table 2).

**Table 2:** Fracture of bones in different circumstances.

Fractures	Assault n (%)	Accident n (%)	Suspicious n (%)	Unknown n (%)	Total n (%)
Fracture of lower limb bones	1 (0.59)	14 (21.54)	0 (0)	0 (0)	15 (6.33)
Cranial bones and cervical vertebrae	1 (0.59)	1 (1.54)	0 (0)	0 (0)	2 (0.84)
Cranial and facial bones	0 (0)	2 (3.08)	0 (0)	0 (0)	2 (0.84)
Shoulder dislocation	0 (0)	1 (1.54)	0 (0)	0 (0)	1 (0.42)
Clavicle, scapula	0 (0)	1 (1.54)	0 (0)	0 (0)	1 (0.42)
Lower Limb and Cervical Vertebrae	1 (0.59)	0 (0)	0 (0)	0 (0)	1 (0.42)
Cranial, Scapula, Thoraco-Lumbar Vertebrae, Ribs	0 (0)	1 (1.54)	0 (0)	0 (0)	1 (0.42)
Clavicle	0 (0)	1 (1.54)	0 (0)	0 (0)	1 (0.42)
Tooth and lower limb bones	0 (0)	1 (1.54)	0 (0)	0 (0)	1 (0.42)
Pelvis and cranial bones	1 (0.59)	1 (1.54)	0 (0)	0 (0)	2 (0.84)
Scapula	0 (0)	1 (1.54)	0 (0)	0 (0)	1 (0.42)
Lower limb and cranial bones	0 (0)	1 (1.54)	0 (0)	0 (0)	1 (0.42)
Lower bone and facial bones	0 (0)	1 (1.54)	0 (0)	0 (0)	1 (0.42)
Upper limb bones	7 (4.12)	5 (7.69)	0 (0)	0 (0)	12 (5.06)
Facial bones	12 (7.06)	0 (0)	0 (0)	0 (0)	12 (5.06)
Upper and lower limb bones	0 (0)	4 (6.16)	0 (0)	0 (0)	4 (1.69)
Cranial bones	9 (5.29)	4 (6.16)	0 (0)	0 (0)	13 (5.48)
Teeth	3 (1.76)	0 (0)	0 (0)	0 (0)	3 (1.26)
Total fractures	35 (20.59)	39(60)	0 (0)	0 (0)	74 (31.18)
No fractures	135 (79.41)	26 (40)	1 (100)	1 (100)	163 (68.82)
**Total**	**170 (100)**	**65 (100)**	**1 (100)**	**1 (100)**	**237**

Most of the individuals presented with injuries over multiple sites of the body 96 (40.5%), 84 individuals presented with injuries overhead and neck (35.4%), similarly 15 (6.3%) individuals had injuries over upper limbs and lower limbs each, 11 (4.6%) had injuries over both upper and lower limbs. Also, injuries overhead the neck were more common in physical assault (46.47%) whereas injuries over limbs were more common in accidental injuries (29.68%). Most physical assaults were under the influence of alcohol 22.94%, in comparison to accidental injuries where only 3.12% were under alcohol influence.

The majority of injuries were non-life-threatening 189 (79.77%), while only 47 (19.83%) were life-threatening. Most of the accidental injuries were found to be life-threatening compared to injuries produced as a result of physical assault (Table 3).

**Table 3:** Life-threatening injuries in different circumstances.

Life-threatening injuries	Assault n (%)	Accident n (%)	Suspicious n (%)	Unknown n (%)	Total n (%)
Yes	18 (10.59)	27 (41.54)	1 (100)	1 (100)	47 (19.83)
No	152 (89.41)	38 (58.46)	0 (0)	0 (0)	189 (79.77)
Total	170 (100)	65 (100)	1 (100)	1 (100)	237 (100)

Whereas most of the physical assaults were non-life-threatening (89.41%) with no involvement of injuries to internal organs, and 27 out of 64 (42.18%) cases of accidental injuries were life-threatening with either fracture or internal organ injuries. Among accidental injuries Road traffic crashes were the most common amounting to 57 (89.06%) cases out of 64 accidental injuries, while 3 cases were due to accidental fall, 2 cases of accidental electrocution, and 1 case of crush injury. The majority of victims of road traffic accidents were pedestrians followed by motorcycle riders (Table 4).

**Table 4:** Victims in a road traffic crash (n= 57).

Victims	n (%)
Pedestrians	21 (36.8)
Motorcycle rider	10 (17.5)
Pellion rider	9 (15.7)
Driver (four-wheeler)	1 (0.17)
Passenger (four-wheeler)	16 (28)

In the case of physical assault, most of the victims were assaulted by fits and kicks 70 (41.17%), while the rest of the victims were assaulted using weapons like stones and bricks in 25 (14.7%) cases, sticks and rods in 24 (14.11%) cases. In 18 (10.5%) cases sharp weapons like knives, khukuri, glass, and axes were used. The cause of violence in case of physical assault were arguments or disputes in 86 (50.5%) cases, financial issues in 10 (5.88%) cases, unknown in 47 (27.64%) cases. The remaining 30 (17.64%) cases were due to causes such as robbery, a relationship issue while separating a fight, and working as security personnel. Most of the victims were assaulted by strangers followed by their neighbors or villagers (Table 5).

**Table 5:** Relationship between victim and assailant in case of physical assault (n= 170).

Relationship	n (%)
Strangers	80 (47.05)
Neighbour/Villager	30 (17.46)
Friends	22 (12.94)
Family members	10 (58.82)
Co-workers	7 (4.11)
Husband/wife	6 (3.5)
Customers	2 (1.17)
Room mate	2 (1.17)
Not mentioned	11 (6.47)

The study showed that most people were prone to get injuries in the street (132/55.7%). This was followed by home 36 (15.2%). Similarly, people also sustained injuries at hotels or restaurants 28 (11.8%) and their workplaces 13 (5.5%). A minority 5% sustained injuries at hostel, hospital, and neighbourhood. In 13 (5.5%) of cases, the place of injury was not mentioned.

Medicolegally injury was classified as grievous and non-grievous as per the criminal code of Nepal^1^ and non-grievous injuries were again differentiated as simple, severe. The study showed that 52 (22%) of the injuries were grievous whereas 185 (78%) were non-grievous (simple 156 (65.8%), severe 29 (12.2%).

## DISCUSSION

The present study revealed that most of the victims of injury are young males, which is similar to studies done in other parts of the country.^4,5^ One of the reasons for this might be due to the male being more active, involved in work and has to travel. A study which was done in 15 different districts of Nepal showed that the mean age of the individual getting injured was 32.6 years,^6^ which is similar to the mean age done in this study 32.41 years. A study done in India also showed that victims of road traffic accidents are mostly at the 3rd decade of life.^5^ This shows that people in their active and productive stage are life are more involved in injuries. This should be a concern as this can result in an economic loss to the community and country as a whole.

Although Unintentional injuries are more common than Intentional injuries (physical assault),^3,7,8^ in our study physical assault outnumbered accidental injuries.

Among the accidental injuries, road traffic crashes were found to be more common in this study (89.06%). In a study done in Nepal, fall injury was found to be more common than road traffic crashes.^4,6^ This discrepancy can be because this study was done in medico-legal cases for injury reports and most of the minor accidental injuries are not reported to legal bodies for the injury report, except in cases of road traffic accidents and occupation-related accidental injuries where the medico-legal report is required for compensation and mostly physical assault is reported to legal bodies even if they are of minor nature.

The most common site of injury in our study was found to be the head and neck region of the body (35.4%). In this study physical assault was more common than accidental injury, and in a physical assault, the perpetrator usually targets the facial region of the body. This result was similar to a study done in other parts of Nepal.^9,10^ In our study extremities were commonly injured in accidental injuries (RTA and Fall) (29.68%) which is similar to a study done by Gupta S, et al. but the proportion was even higher (68.6%).^6^

Most of the accidental injuries were associated with bone fractures (60.93%) whereas the majority of physical assault was not associated with bony fractures (79.4%). Similarly, fractures of the extremity were more common in accidental injury (46.87%) and fractures of facial bones were relatively more common in physical assault (8.82%). Most of the accidental injuries were life-threatening with injury to internal organs mostly associated with a head injury, indicating that accidental injuries were more violent and severe than physical assault. This result was supported by a study that showed that most of the RTA cases had head injuries (43.22%) and head injuries were significantly associated with fatality.^5^

In this study, most of the injuries were sustained on the street (55.7%), for both accidental injuries and physical assault, this was consistent with another study done in Nepal, which also showed that most injuries were sustained at streets or highways.^4^ In cases of physical assault the most common reason for violence being dispute or arguments followed by monetary issues. Most of these arguments were under the influence of alcohol (22.94%). However, road traffic crashes under the influence of alcohol were very low in our study (3.12%) probably because of the strict law of our country that prohibits driving under the influence of alcohol. This result was in contradiction to studies done by other authors, who found alcohol consumption in the majority of cases of road traffic crashes (15-46%).^5,11^ The proportion of alcohol consumption in road traffic crashes should be higher than it was recorded in the study, as it was based on the history provided by the victims. If blood or urine for alcohol were tested in these cases the proportion could be even higher.

In this study among the road traffic crashes, pedestrians were the common victims, which was similar to a study done in other parts of Nepal.^4,9^ This could be due to a lack of public awareness among people walking on the road. However, in a study done in the western part of Nepal vehicle passengers were more commonly involved in road traffic crashes (42.5%) and pedestrians were 2nd most common victims (29.16%).^5^

In cases of Physical assault, most victims were attacked by fists and kicks. This was consistent with a study done in eastern Nepal.^10^ The choice of weapons also included household items like lock pads, helmets, glass, stones, and bricks (21.75%), indicating that the assault was not pre-planned. In few cases weapons like sticks, sharp instruments (10.5%) like khukuri, knives were used, indicating planned assault. In a study done in the western part of Nepal, it was found that wooden sticks and clubs were common than (21.5%) kicks and punches (20.6%).^9^ Even in developed countries line USA fist and kicks are most commonly used during an assault.^12^

The study showed that 22% of the injuries were grievous and 78% were non-grievous. The incidence of grievous injuries is even less in other studies done in Nepal.^10^ In this study most of the injuries were non-grievous and it was further sub-divided as simple and severe injuries as non-grievous injuries were a legal term described in the criminal code of Nepal but medically these non-grievous injuries were still severe enough to cause a threat to life.

This study has limitations. This study is done in a single center and only includes cases referred by police for preparation of medicolegal injury reports.

## CONCLUSIONS

The prevalence of injuries amongst medicolegal cases was found to be higher in our study in comparison to other studies done in similar settings. Most of the injuries were due to physical assault; however, the majority of road traffic injuries were life-threatening. The majority of these injuries could have been prevented by various measures like educating about road safety, following the safe system approach by the government of Nepal that includes the components such as good road safety management, safer roads, safer vehicles, safer road users, and a proper post-crash response.^13^ Since recreational substances like alcohol are also involved in most cases proper and responsible use of these substances should be promoted.
